# Three nested RCTs of dual or single stakeholder feedback within Delphi surveys during core outcome and information set development

**DOI:** 10.1186/1745-6215-16-S2-P51

**Published:** 2015-11-16

**Authors:** Sara Brookes, Rhiannon Macefield, Paula Williamson, Angus McNair, Shelley Potter, Natalie Blencowe, Sean Strong, Jane Blazeby

**Affiliations:** 1 University of Bristol, Bristol, UK; 2 University of Liverpool, Liverpool, UK; 3 University Hospitals Bristol NHS Foundation Trust, Bristol, UK

## Background

In the development of a core outcome or information set the Delphi technique requires participants to rate the importance of items in sequential questionnaires (rounds). Feedback provided in each subsequent round enables participants to consider views of others. This paper examines the impact of separate stakeholder group feedback.

## Methods

Two rounds of Delphi questionnaires were completed by patients and health professionals during the development of three surgical core sets. Participants rated items 1 (not essential) to 9 (absolutely essential). For round 2, participants were randomized to receive feedback from their stakeholder group only (single) or both stakeholder groups separately (dual). Items to retain following each round were determined by pre-specified criteria.

## Results

Type of feedback did not impact on the proportion of items where a participant changed their rating, nor the magnitude of change. Items retained after round 2 were similar between the feedback groups, but each core set contained discordant items retained by one feedback group and not the other (kappa 0.44 to 0.91). Consensus between patients and professionals in items to retain was greater amongst those in the dual group in each core set. Differences and variability in round 2 scores were smaller between stakeholders receiving dual feedback (P≤0.02 for all).

## Conclusions

In the development of a core outcome or information set, providing feedback within Delphi questionnaires from each stakeholder group separately may influence the final set and improve consensus. Further work is needed to better understand how participants rate and re-rate items within a Delphi process.

